# A Review on Recent Advances of Cerebral Palsy

**DOI:** 10.1155/2022/2622310

**Published:** 2022-07-30

**Authors:** Sudip Paul, Anjuman Nahar, Mrinalini Bhagawati, Ajaya Jang Kunwar

**Affiliations:** ^1^Department of Biomedical Engineering, North-Eastern Hill University, Shillong 793022, India; ^2^Department of Anatomy, Nepalese Army Institute of Health Sciences, College of Medicine, Kathmandu, Nepal; ^3^Kathmandu Center for Genomics and Research Laboratory, Kathmandu, Nepal

## Abstract

This narrative review summarizes the latest advances in cerebral palsy and identifies where more research is required. Several studies on cerebral palsy were analyzed to generate a general idea of the prevalence of, risk factors associated with, and classification of cerebral palsy (CP). Different classification systems used for the classification of CP on a functional basis were also analyzed. Diagnosis systems used along with the prevention techniques were discussed. State-of-the-art treatment strategies for CP were also analyzed. Statistical distribution was performed based on the selected studies. Prevalence was found to be 2-3/1000 lives; the factors that can be correlated are gestational age and birth weight. The risk factors identified were preconception, prenatal, perinatal, and postnatal categories. According to the evidence, CP is classified into spastic (80%), dyskinetic (15%), and ataxic (5%) forms. Diagnosis approaches were based on clinical investigation and neurological examinations that include magnetic resonance imaging (MRI), biomarkers, and cranial ultrasound. The treatment procedures found were medical and surgical interventions, physiotherapy, occupational therapy, umbilical milking, nanomedicine, and stem cell therapy. Technological advancements in CP were also discussed. CP is the most common neuromotor disability with a prevalence of 2-3/1000 lives. The highest contributing risk factor is prematurity and being underweight. Several preventions and diagnostic techniques like MRI and ultrasound were being used. Treatment like cord blood treatment nanomedicine and stem cell therapy needs to be investigated further in the future to apply in clinical practice. Future studies are indicated in the context of technological advancements among cerebral palsy children.

## 1. Introduction

Cerebral palsy is the most common disability of childhood that affects motor function as a result of injury to the developing brain [1]. It is also known as Little's disease as the term was first described by William John Little in the year 1843 in which he mentioned spasticity occurs due to damage to the brain during infancy, preterm birth, or birth asphyxia. This was followed by extensive contributions of Osler, Sach, and Peterson, Sigmund Frued, Mac Keith and Polani, and many others until 2006 when an expert executive panel defined CP as a group of permanent disorders of the development of movement and posture, causing activity limitation, which is attributed to nonprogressive disturbances occurring in the developing fetal or infant brain. CP symptoms are heterogeneous, a child having limited brain injury may find difficulty in just one component of the musculoskeletal system, and another child with a broad range of symptoms may suffer from activities that hamper the activities of daily living of the child along with other life-threatening comorbidities; however, its symptom may improve in due course of time owing to the maturity of the nervous system with age. Damage to the developing brain before, during, or just after delivery affects both neurological and musculoskeletal systems of the body producing symptoms such as abnormal contraction of muscles, postural changes, and movement and activity limitation which are accompanied by sensory disturbances along with perceptual disorders, cognitive issues, inability to communicate, behavioral issues, epilepsy, and secondary musculoskeletal problems. Previously, it was thought that lack of oxygen at birth is responsible for cerebral palsy; however, with emergent research, it is evident that along with this, there are many other causes and risk factors responsible for the development of cerebral palsy. It is now believed that CP results from a series of events that combines to cause injury to the brain during the developmental period [2, 3].

The epidemiology of CP has changed over time. It occurs in 2-3 in every 1000 live births; however, it is relatively stable over decades [1]. The prevalence of CP was found to be in increasing trend in studies done before 1990 as there was better survival of preterm infants due to advances in medical technology; however, the prevalence decreased subsequently as there was an improvement in prenatal care too after that. The prevalence has remained the same from 1990 to 2003 and was found to be between 2.2 and 2.3; however, it has decreased now [2, 4]. According to another study, the prevalence of CP has decreased from 2.1 to 1.4 in Australian children since 1995; apart from these, various studies show that due to the financial burden of the developing countries, children cannot get the best service for prevention and management of CP which has led to its increased severity; these population trends indicate that changes in preventive and management studies are successful; however, more research is required in these areas. Increasing evidence of various low-cost novel treatment techniques that can be made easily available to the population promises to deliver better outcomes [5, 6]. The incidence of CP is found to be stable in worldwide epidemiological studies, but the management of premature birth complications is still a contributory factor in increasing the incidence of this disease [7]. In the last decade, various prevention and management strategies have been identified in the literature that helped in decreasing the occurrence of this disease. Prescribing magnesium sulfate, progesterone, and corticosteroids to pregnant women for their neuroprotective nature and application of therapeutic hypothermia are some of the evident methods of preventing prematurity which is a major causative factor of CP [5, 6]. This review is aimed at summarizing the strategies that will improve the status of children with CP. The review will begin by compiling research done on the risk factors and etiologies of CP in the last 5 years including efforts to standardize diagnostic criteria along with classification and clinical features of CP. The prevention and management strategies will then be focused on in detail.

## 2. Methodology

The study is aimed at throwing light on recent developments in cerebral palsy and showing new paths for future research in this field. For this purpose, various types of published articles including original research, review articles, and systematic reviews that we consider relevant to our study were selected. A search in Medline via PubMed, Google Scholar, and manually extracted relevant publications by cross-referencing was done to find publications made in the English language. Search terms included cerebral palsy or early brain lesion or perinatal stroke along with management and prevention techniques. We focused on publications of the last five years, i.e., 2017 to 2021, to provide an updated overview.

## 3. Results

### 3.1. Risk Factors for and Etiology of Cerebral Palsy

**Figure 1:**
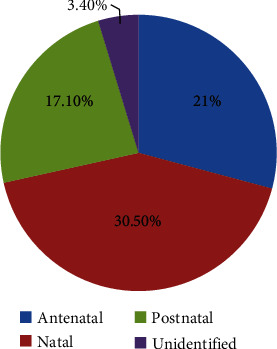
Risk factors for cerebral palsy [2, 7, 10].

It is now well known that the prime risk factors for CP are delivery before 37 weeks and birth weight of less than 2.5 kg; however, there are some other problems evident in the literature which are found to be some of the prominent reasons for brain damage, some of which includes malformation of the brain in the developmental period, genetic causes, in utero mother and fetus infections, and various other issues [8]. Factors that may put the developing brain prone to injury were divided into risk factors that develop during preconception, during pregnancy, and after birth [2]. A study confirmed that the health of the mother before conception is one of the reasons that affect the central nervous system of the fetus later during the gestational period which might lead to CP. According to this study, preconception is defined as the health conditions of the mother before conception, prenatal is defined as the period of gestation, perinatal is during delivery, and postnatal is after delivery [2]. A study in 2021 also found that the risk factors for cerebral palsy were 21%, 30.5%, 17.1%, and 31.4% when grouped under prenatal, perinatal, postnatal, and unidentified categories [9]. Preconception risk factors include the mother's systemic illness, substance abuse, maternal undernutrition, swallowing harmful substances, fertility issues, and previous spontaneous termination of pregnancy [7]. Factors that may lead to brain damage during gestation include maternal abnormalities of the central nervous system, gestational diabetes, excessive bleeding per vagina, and preeclampsia. Multiple gestations, cotwin death, genetic contributions, and encephalopathy of prematurity are also strong risk factors for CP [7]. Risk factors during delivery are premature birth, C-section, vacuum-assisted delivery, forceps delivery, delivery after the due date, labor induction, prolonged labor, asphyxia, and meconium aspiration syndrome [10]. Various other risk factors before, during, and after delivery that may lead to brain damage are summed up in Table 1 and Figure 1.

**Figure 2:**
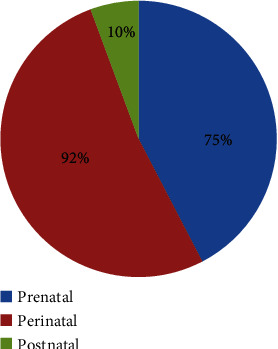
Causes of cerebral palsy [2, 8, 11–13, 15].

**Figure 3:**
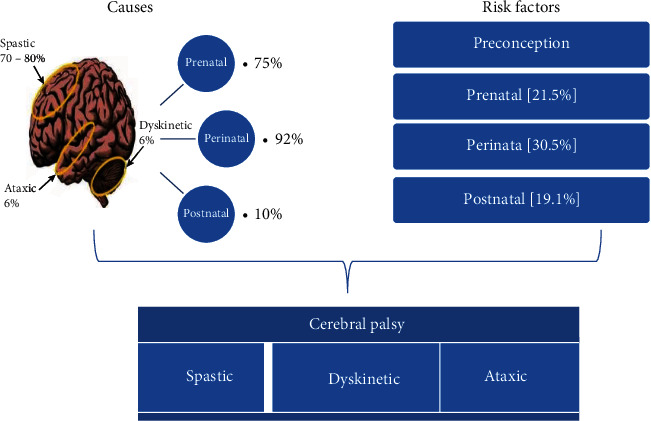
Events leading to cerebral palsy [2, 8, 11–13, 15].

Multiple etiologies are responsible for various developmental defects in the fetal brain that results in brain injury which affects the physical functioning of the body [11]. Nearly 75% of CP occurs due to prenatal etiologies whereas 92% of causes are perinatal [2, 12]. It is now well known that CP results from various reasons during pregnancy or during delivery, but in various studies, it is found that it occurs due to brain injury in the postneonatal period also [8, 12]. Postnatal CP is defined as any trauma or disease in the brain after a neonatal period and before 5 years of age [13]. Immediately after delivery, CP may occur in 10-18% of cases due to conditions like hypoglycemia, jaundice, and infections [2, 12, 14, 15]. Though preterm is considered an important benchmark in the etiology of CP, term babies are also high in percentage; this might serve as an indication of a genetic basis associated with CP [2]. For term-birth children, pieces of evidence also suggest that sudden genetic mutations in genes may also be responsible for the development of CP without any other probable causes [16]. Placenta abruption, prolapsed cord, birth asphyxia, congenital anomalies, and maternal conditions during labor like high fever are common causative agents that may lead to brain damage in the fetus. Congenital etiologies such as failure of closure of the neural tube, schizencephaly, chromosomal defects, and microcephaly are also some of the causes [8]. In children born in less than 32 weeks, white matter injury in 84.6% of children was evident which was found to increase with decreasing gestational age [13, 16]. Grey matter injury was seen in moderately preterm infants. Bilateral CP was found to be in a higher percentage than unilateral CP with a decrease in gestational age [15]. A study in 2019 among 2-15-year-old children in Nigeria revealed that most cases were due to birth asphyxia, hyperbilirubinemia, and rubella [2]. An SCPE collaborative study in 2021 referred to the common causes of various types of CP as PVL, congenital infections, asphyxia, hyperbilirubinemia, genetic, neonatal stroke, etc. [15]. Various other causes of CP are listed in Table 2 and Figure 2. Apart from this, events that lead to CP are demonstrated in Figure 3.

### 3.2. Classification of Cerebral Palsy

**Figure 4:**
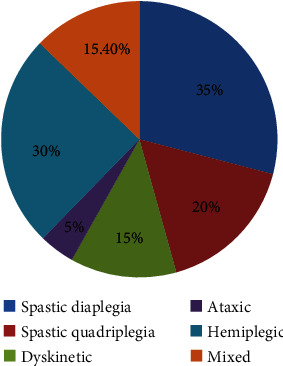
Different types of cerebral palsy [2, 14].

As injury to the developing brain occurs due to numerous causes and manifests in different clinical presentations and severity, it has been described under various headings based on the type of movement disorder, area of involvement, and level of damage. According to the type of movement disorder, CP is classified as spastic, dyskinetic, and ataxic. Based on the area of presentation, it can be classified into involvement in one side or both sides of the body, i.e., quadriplegic, hemiplegic, diplegic, and monoplegic, diplegic being most common followed by hemiplegic (20–30%) and quadriplegic (10–15%) (Figure 4). In quadriplegic CP, all four limbs are affected. In this condition, the hands are more affected than the legs, and this occurs due to acute hypoxic asphyxia during the perinatal period, excessive cystic degeneration of the brain, and developmental abnormalities such as polymicrogyria and schizencephaly. The condition presents with limited voluntary movements of all the extremities, pseudobulbar signs, accidental food entry in the airways, difficulty in swallowing, optic atrophy, seizures, and severe intellectual abnormality. In hemiplegic CP, hand functions are mostly affected. Dorsiflexion and aversion of the foot are severely impaired in the lower limb. Increased spasticity in flexor muscles, sensory abnormalities, seizures, and visual problems are common findings. In diplegic, CP cystic periventricular leukomalacia is the most common neurological feature seen in premature infants. In the case of hemiplegic CP, only one side of the body is affected with a high tone in flexor muscles and sensory loss. Apart from this, hand function is severely impaired when compared to legs. In the foot, dorsiflexion and eversion are affected. Both matured and premature-born children are at risk of hemiplegic CP [14].

Popularly, CP was classified according to the Ingram and Hagbergs classification; however, surveillance of cerebral palsy in Europe (SCPE) has simplified the classification of CP as spastic, ataxic, and dyskinetic (Table 3) [2]. Ingram classified CP based on location and severity of neurological symptoms. He classified cerebral palsy into diplegic, hemiplegic, tetraplegic, ataxic, dyskinetic, and mixed [21]. Hagberg however classified CP into spasticity, dyskinetic syndromes, and ataxia. The spastic syndrome occurs due to damage to the brain and tracks controlling movement. It can be divided into monoparesis, hemiparesis, triparesis, tetraparesis, and spastic diplegia. Dyskinetic symptoms are seen due to injury to the subcortical structure, and ataxic symptoms are seen due to cerebellar injuries dividing CP into spastic, affecting one or both sides of the body, dyskinetic involving involuntary movements with altered tone or choreoathetosis movements, and ataxic. Around 80% of CP cases are found to be spastic [22]. Spastic CP in this context is characterized by increased muscle tone and increased reflexes. It has been subcategorized into unilateral or bilateral along with the area of involvement. SCPE refers to dyskinetic CP accounting for 10 to 20% of CP cases and presents as having involuntary, uncontrolled, repetitive, and sometimes stereotypical movements with a fluctuating muscle tone. A faulty posture with enhanced muscle tone is defined as dystonic; a quick, uncontrolled, and twisting movement with hypotonia is called choreoathetosis. On the other hand, ataxic CP consists of 5-10% of CP cases and presents with loss of coordination with hypotonia. In some children, damage may occur in different parts of the developing brain which causes them to develop symptoms of having a combination of two or more types of cerebral palsy. This type is called mixed CP which accounts for 15.4 percent of all cases. The most common presenting symptoms of mixed type are a combination of spastic and athetoid features [13].

Evaluating the severity of motor disorders is important for predicting the functioning of the affected limbs and the outcome of the treatments. For this purpose, four systems are used for functional classification of CP which include GMFCS, MACS, CFCS, and EDACS [2, 11, 12, 23]. GMFCS developed by Palisano et al. in 1997 is used worldwide for the functional classification of CP (Table 4) [2, 11, 12, 23]. It is easy to use and describes gross motor function. It has levels that describe voluntary movement and the use of aid for movement. It was first designed to measure gross movements in children of 2–12 years of age, but in 2007, it was revised and ages 12–18 were included. According to the new revised version of GMFCS, a child is considered to be in GMFCS level 1 if the individual can walk without any aids. However, there are considerations and limitations according to the age of the child. In level 2, the child can do all these activities, but limitations are present in the form of speed, balance, and endurance. The child finds difficulty walking long distances and requires a handheld or wheeled device for long distances. Gross motor skills are minimal. Level 3 children walk with handheld mobility devices in indoor settings, need supervision during stair climbing, and require wheeled devices for long distances. In level 4, the child lacks self-mobility. The child can sit with support, but transportation requires a manual or powered wheelchair. In level 5, children are dependent on all settings and have limitations to maintain antigravity posture. They strictly require wheelchair transportation. Another classification system, namely, the manual ability classification system (MACS), is a five-level scale used for 4–18 years old which was developed by Eliasson et al. in 2006, to evaluate the functions of the upper limb. In level I, the child can handle objects with ease, there are some limitations with accuracy, but that does not hamper activities of daily living. In level II, the child's activities are slower and of reduced quality. A different way to perform the activity can be used by the child, but it does not affect the daily activities performed by the child. The child in MACS III has reduced speed while performing hand activities and often with limited success. Some activities need help, but others can be done without any help. A child in MACS IV performs various simple activities with lots of effort. They require constant help and adapted types of equipment for performing simple activities. Individuals in MACS V are dependent [24].

A classification system, namely, the communication function classification system (CFCS), is also a five-level scale that is used to evaluate everyday communication. At an individual level, level I can communicate at a comfortable pace. The person can send and receive information from different people in different individuals. In level II, the pace of communication is slow; however, they can communicate properly. In level III, communication is effective only with a familiar partner. In level IV, the person is not always consistent in communication with known people, whereas in level V, the individual cannot communicate affectively and consistently with unknown people. The eating and drinking ability classification system is again a five-level classification system used to assess how efficiently a CP child eats and drinks. It is used for more than 3-year-old children. It has extra three levels that help to find out how much help is required while performing these activities. In EDACS level I, an individual can eat and drink safely without any help, but hard food can cause difficulty swallowing. In level II, the individual can eat and drink safely but has very less speed. The child may present with a cough when food is given at an increased speed. In level III, the child cannot eat hard food; he/she needs very soft and mashed food. However, an individual in EDACS IV or V cannot swallow food and drink safely. Tube feeding is required to provide nutrition [24].

### 3.3. Clinical Presentations of Cerebral Palsy

**Figure 5:**
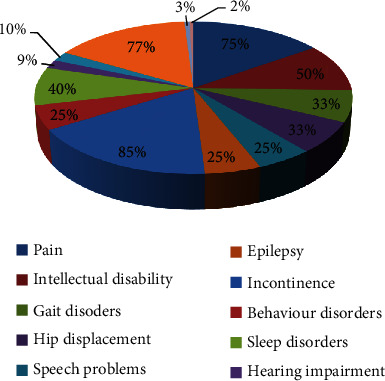
Comorbidities associated with cerebral palsy [11, 17–20].

The presenting signs and symptoms of CP are diverse and mainly consist of motor disorders, sensory deficits, and associated comorbidities which occur due to a static lesion to the developing brain. These signs and symptoms change as the child ages and new features are added to the list. Thus, with advanced age, there is a worsening of the neuromuscular system and functional capability of the child even though the damage in the brain is static [25]. Injury to the fetal brain can be generally diagnosed by its presenting features; however, often, some of these symptoms resolve after 2 years in many infants owing to the maturation of the CNS [12]. A recent study revealed that the most common symptoms seen were using one hand before 2 years of age, inability to reach normal milestones within the appropriate time, and presence of primitive reflexes after a definite period (Table 5) [22]. Some comorbidities are also associated with cerebral palsy which are summarized in Table 6 [11, 17–20] and demonstrated in Figure 5 [11, 17–20]. Hypertonicity of the muscles owing to brain injury is the most common symptom seen in CP patients along with other motor issues such as impaired balance, coordination, hand function, etc. [10]. A recent study found that this may be due to three causes, i.e., more muscle fibers are required to perform a certain task than healthy individuals, excessive level of collagen deposition in myofibers decreases the efficiency of the muscles by making them stiff, and a disturbance in the neuromuscular junction causes a problem in muscle contraction. The study also revealed that collagen accumulation occurs due to damage to CNS in the developing brain, and this causes issues in the motor abilities of the child as mentioned above. However, there is scarce evidence on the prevention and treatment of this finding [25].

Spastic diplegia is the most common type that accounts for 35% of cases and occurs due to damage to the immature oligodendroglia in the second trimester. In 3- to 6-month-old babies, some of the features seen are decreased neck control, stiffness, floppiness, arching of the back, lower extremity stiffness, and leg crossing while raising from the bed, and in babies older than 6 months of age, there is no rolling. Incoordination of the upper extremities is also evident. Babies older than 10 months of age depict abnormal crawling. Periventricular leukomalacia is the most common neurological finding seen in such cases. Another type of spastic CP apart from diplegic is spastic quadriplegia which accounts for 20% of CP children; the most common reason is premature birth. The child has severe motor and sensory problems, cognitive deficit, seizures, vision problems, and other associated problems which make the child completely dependent. Term infants who are at risk of in utero or perinatal stroke suffer from spastic hemiplegia. They have good cognition and can maintain independent mobility. 15% of CP results from extrapyramidal lesions in term babies. They consist of involuntary movements termed choreoathetosis, dystonic, or dyskinetic clinical features. Hemiplegic CP cases are mostly term babies having causes like brain injury due to lack of oxygen, kernicterus, and neurometabolic or genetic disorders [2]. A high mortality rate is seen in CP children due to respiratory problems [26].

### 3.4. Diagnosis

**Figure 6:**
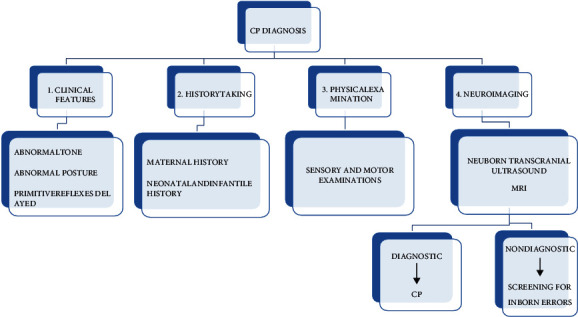
Diagnostic criteria for detecting cerebral palsy [2, 13].

Early diagnosis is necessary as it helps to provide early intervention during the earliest period of development. It is a special service to prevent developmental delay which optimizes the impact of the interventions on the developing brain's neuroplasticity [4, 27]. Diagnosis of cerebral palsy is based on the combined use of clinical presentations along with physical assessments and neuroimaging, which can provide various implications for this disease. Assessment of maternal history including the child's performance of motor functions brings out important points of diagnosis. Owing to the complexity of the condition, psychological tests, vision evaluation, audiometric tests, and electroencephalography are carried out [2]. Close monitoring of early signs in the form of neurobehavioral signs, presence of developmental reflexes that did not disappear with time, abnormal tone and posture, and delayed milestones along with associated comorbidities is essential to screen risk infants. The history of early diagnosis started in the 1800s when William Little urged that the earliest diagnosis will lead to early intervention. It is very important to find out the cause of CP and give the required treatment so that the disease process can be minimized along with increased neuroplasticity and functional outcome. In the 1970s, the idea of risk factors, retaining of abnormal primitive reflexes, and the cranial US in neonatal intensive care units were introduced which helped to identify children who were at risk for CP. In high-income countries, diagnosis of CP was previously done after 1 year, but now, it can be done before 6 months [4]. In 2017, a systematic review was published which inferred that certain tools can be used to diagnose high-risk infants for the development of CP as early as 6 months. A list of such tools is given in Table 7. Prechtl's Qualitative Assessment of General Movements and the Hammersmith Infant Neurological Examination can also be used as predictive tools along with clinical examination in infants below 5 months of age. After 5 months, magnetic resonance imaging, the Hammersmith Infant Neurological Examination, and the Developmental Assessment of Young Children are used to predict CP in extremely low birth weight infants [4]. A combination of two MRI biomarkers fractional anisotropy of superior thalamic radiations and radial diffusivity of the corticospinal tract was used to evaluate the brain's sensory and motor tracts, respectively [5]. In 2011 a study named “cerebral palsy—don't delay” summed up the call for early detection and accurate prediction of CP in the earliest months of life by referring to general movement assessment (GMA), first introduced in 1990 which is an assessment of the spontaneous movement of an infant along with another standardized neurological examination called the Hammersmith Infant Neurological Examination (HINE) [4]. In 2019, a paper was published on the topic of international expert recommendations of clinical features to prompt referral for diagnostic assessment of cerebral palsy where a survey was conducted among 51 international experts in Asia, USA, Australia, Canada, and Europe to find out agreement upon early motor signs and diagnosis of CP and their referral to other health professionals under a project called PROMPT (primary care referral of motor impaired children). The international experts provided a strong agreement on six clinical features and two warning signs along with five referral recommendations based on which a child should be immediately referred for diagnosis to other health care professionals or specialized health services [28]. The American Academy of Neurology recommends a stepwise protocol to help diagnose a cerebral palsy child. The first step is the recognition of the disease by clinical history taking and physical examinations followed by screening for associated comorbidities. This is followed by studying perinatal histories such as fetal anatomy surveys and newborn transcranial ultrasounds. If no abnormalities are detected, MRI is recommended for finding out intracranial abnormalities. Further, if the test is nondiagnostic, then screening for inborn errors of metabolism or genetic abnormality is followed [13]. The entire process is described in Figure 6 [13]. Based on some studies done on dead CP children, Little found that there is some venous and capillary congestion in the brain and spinal cord which led him to refer to this disease as a cerebrospinal disorder. However, William Osler was the person who gave the name cerebral palsy to this condition. Though modern definitions of CP are refined to the context of the cerebral cortex, a critical evaluation study on the concept of CP urges that more studies should be done on the original concept of CP as a “cerebrospinal” disorder, both in clinical work and in animal models [29].

### 3.5. Prevention

**Figure 7:**
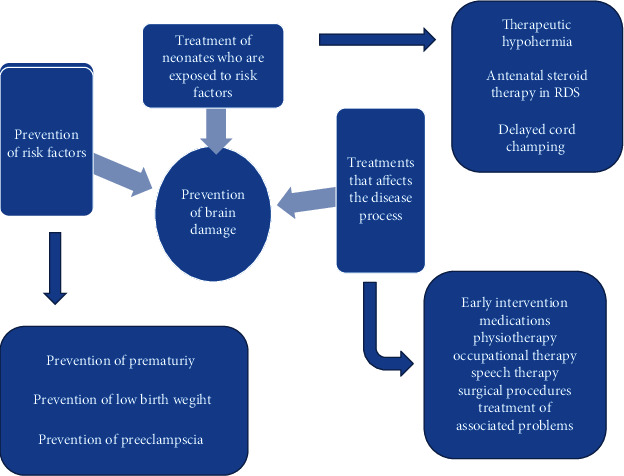
Prevention and management of cerebral palsy [28, 30, 31].

Based on the time of insult to the brain, CP can be divided into individuals whose brain injury occurred during the gestational period, during delivery, and postdelivery. Thus, prevention strategies can depend on the prevention of factors that will decrease the risk of CP in the antenatal, perinatal, and postnatal periods [30]. Prevention strategies include prevention of risk factors, treatments that affect the disease process, and treatment of neonates who are exposed to risk. Various techniques in the literature are present for the prevention of brain injury during the gestational period and delivery. Administration of magnesium sulfate is an important preventive measure for high-risk mothers [28, 31]. There is moderate quality evidence of an increased rate of CP in mothers who used prophylactic antibiotics during pregnancy. Latest reports show that prenatal and perinatal causes of CP have decreased. This has occurred due to various strategies that are used for the early treatment of neonates [30]. The worldwide incidence of birth before 37 weeks is 12% and is one of the main causes of death and illness in neonates. Various studies infer that prophylactic use of progesterone decreases early birth in women with previous birth complications. Universal cervix screening is recommended by midtrimester transvaginal ultrasonography. Management of IUGR, administration of magnesium support, and corticosteroids for fetal lung maturity are equally important strategies [7, 32]. Decreasing the rate of early birth and low weight in neonates is the most significant consideration in reducing the overall incidence of CP. Therapeutic cooling or hypothermia is helpful in cases of brain injury due to a lack of oxygen. It decreases the risk of CP in term and late preterm infants in such cases. It is started within 6 hours after birth which helps to decrease the temperature by 2°C for 48 hours [28, 32, 33]. Prevention of preeclampsia is done by screening and administration of acetylsalicylic acid along with aspirin which should be started before 16 weeks of gestation, with a daily dose higher than 100 mg in high-risk patients. This issue, however, requires further prospective research. Antenatal steroid therapy is evident in newborns in preventing perinatal death of newborns and preventing the risk of disability and development of sepsis in the initial days of birth. Delayed cord clamping is another intervention used in preterm babies that lowers the risk of bleeding, necrotizing enterocolitis, and anemia that requires blood transfusion and late onset of sepsis which has an impact on the neurological development of the baby. Preventive techniques during pregnancy also include corticosteroids for the mother for accelerating lung maturation in the case of early-birth infants. The literature also has evidence of caffeine for apnea of prematurity. Apart from preterm infants, those who are born at the expected time and suffer from a lack of oxygen during delivery have also benefitted from therapeutic hypothermia. Factors that can prevent postnatal CP that are evident from the literature include finding hidden cases of group B streptococcus, administration of antibiotics and vaccines during and after delivery, safe car seating, safety measures in swimming pools, and preventing shaking of the baby [30]. As CP is seen to occur mostly due to prenatal causes (45%), preventing strategies during this period can decrease the overall incidence of CP. To implement this, a recent study in 2018 advised certain interventions to reduce preterm birth: midwife-led continuity models of care, finding and treating urinary tract infections, augmenting zinc supplementation among pregnant women, and cervical cerclage for high-risk mothers [30, 34]. A schematic diagram for the prevention of cerebral palsy is given in Figure 7.

### 3.6. Management

Cerebral palsy management is aimed at improving functional ability and independency and managing secondary complications. Physical and occupational therapies, mechanical aids, orthopedic surgery to address patients' motor problems, and optimal medical and surgical treatment of medical comorbidities are the main management strategies [35]. An increase in neonatal care and decreased prevalence showed a promising impact on early diagnosis [4]. Early intervention programs are the most essential component of the management of CP as it addresses the disease process at the earliest and helps in early neuroplasticity of the brain [36]. Two trials, namely, GAME (goals, activity, and motor enrichment) and REACH (rehabilitation early for congenital hemiplegia), are under investigation in Australia to establish evidence for early intervention in children with CP [37]. Addressing functions like physical issues, cognition, communication, eating and drinking, vision, and sleep helps in improving the overall health of the child, and cooperation of the family and environment modification are the major factors for improvement [38]. Management of CP children requires a team approach which includes a list of multidisciplinary team members such as an audiologist, medical social worker, nurse, nutritionist, occupational therapist, pediatric gastroenterologist, pediatric neurologist, pediatric orthopedic, surgeon, pediatric pulmonologist, pediatric surgeon, pediatrician, physiatrist, physiotherapist, psychologist, speech-language therapist, and special educator [10]. Many recent advances in the management of CP have come up including intrathecal baclofen, selective dorsal rhizotomy, and sensory integration [14]. Various medical managements are effective in the treatment of associated problems in CP children such as multilevel surgery for epilepsy, benzhexol hydrochloride for saliva control, and laxatives for constipation [27]. NSAIDs reduce pain; gabapentin is effective in dystonic CP [39]. Certain treatment exposures such as cooling, umbilical cord blood treatment, glial cell transplantation, nanomedicine, and stem cell therapy are under investigation and extremely reviewed [14]. Therapies such as physical, occupational, speech, and behavioral therapies help in enhancing patient and caregiver interactions while providing family support [27]. Various novel techniques such as telemedicine with outreach programs of physiotherapy services are found to be beneficial [40].

#### 3.6.1. Spasticity Management

The most common movement disorders seen in cerebral palsy are spastic muscles and dystonia with difficulties in coordination, strength, and selective motor control. Spasticity is the major challenge in the management of CP children. It causes spasticity-induced bone and joint deformity, pain, and functional loss [10]. Commonly used medicines found in the literature to relieve spasticity are baclofen, diazepam, clonazepam, dantrolene, and tizanidine. Baclofen and diazepam help in relaxing the muscles but have many side effects [27]. First-line treatment for spasticity is physiotherapy, occupational therapy and botulinum toxin injections, selective dorsal rhizotomy, and intrathecal baclofen [8]. A selective dorsal rhizotomy is a surgical procedure that is effective in CP children that improve their walking ability and range of movement. It rectifies the spasticity that impairs gait by improving the ankle joint junctions [41]. Administration of intrathecal baclofen is done via an implantable pump. It is reserved for GMFCS levels IV and VI as it is used in extreme spasticity cases. However, it is expensive, and relief is of short duration. Intramuscular onabotulinum toxin type (Botox) weakens the skeletal muscles by impairing the release of neurotransmitters at the NMJ. This slows down the contraction of skeletal muscle. The injection is first given between 18 and 24 months of age [8, 10]. Surgical management including lengthening of the soft tissues such as adductors and hamstrings, multilevel surgery of the ankle and foot, nerve blocking, tendon transfers, and joint stabilization are some of the surgical techniques used in CP age appropriately [27].

#### 3.6.2. Management of Balance and Movement Disorder

Balance and movement disorders are crucial management issues in CP children as they are necessary for maximum activities of daily living. The traditional management approach in CP includes physiotherapy, occupational therapy, hyperbaric oxygen therapy, sensory integration, NDT, hippotherapy, CIMT, BWSTT, acupuncture, and the Vojta method [8, 42]. Two treatment techniques called whole-body vibration along with core stability exercises are found to be effective in managing balance issues, the former being more effective [43]. Another therapy having prospects of improving balance is virtual reality. Giving it for 20 minutes, twice a week for 6 weeks gives very good results in balance improvement [44]. Nintendo Wii therapy is another balance-improving treatment that can be considered an effective treatment for improving functional and dynamic balance. It can be combined with physiotherapy techniques for 30 minutes for a minimum of 3 weeks for effective results [44]. Management of movement disorders however for CP patients includes trihexyphenidyl, tetrabenazine, baclofen, levodopa, benzodiazepines, and deep brain stimulation [45].

#### 3.6.3. Management of Hand Dysfunction

Injury in the brain may cause disturbances in hand functioning which may be unilateral or bilateral. In the former case, motor control and function on one side of the body are affected. In this type of CP, children experience difficulties using their hands on the affected side. Constraint-induced movement therapy (CIMT) is a technique that is being used to improve the function of the affected hand. It is based on the principles that not using the good hand and intensive use of the affected hand improves hand function by neuroplasticity of the brain. A study reveals that the therapeutic effect of CIMT is independent of age. No differences were found between boys and girls for this therapy. CIMT is found to be effective in the literature to improve hand function; however, its effect on muscle tone and protective extension is yet to be investigated. Hand-arm intensive bimanual therapy is a similar technique that improves hand function; however, both hands need to be used in this technique. In a study of children with hemiplegic cerebral palsy, it is found that both of these strategies are promising techniques to improve hand function; however, the latter is more tolerable in children than CIMT. CIMT has also shown improvement in somatosensory functioning and neural processing in such children [46–49].

#### 3.6.4. Management of Hip and Ankle Deformities

36% of CP children suffer from hip disorders which lead to problems such as dislocation, subluxation, and other related problems which can be managed surgically. Hip surveillance programs are recommended to screen cases of hip deformities [40]. In younger children who cannot walk due to hip disorders, reconstructive procedures are useful as they provide long-term results; in cases of degeneration of the hip, reconstruction surgeries such as osteotomy or arthroplasty are done [41]. In ankle equines, the deformity is seen in cerebral palsy children. Orthotic devices can help improve the ankle range which is beneficial in improving the gait of the child. Specific types of AFOs improve joint function and gait parameters. AFOs reduce energy expenditure in children with spastic CP. The HKAFO is very helpful in improving gait parameters and is evident in energy conservation in hemiplegic CP children. Further studies are required for better evidence regarding this [50–52].

### 3.7. Management of Associated Problems of CP

#### 3.7.1. Epilepsy

Epilepsy is a common comorbidity seen in CP children. Children who are grouped under the quadriplegic category with microcephaly often present with seizures. Various systemic and postzygotic genetic mutations are thought to be responsible for neonatal epilepsies. Levetiracetam, valproic acid, topiramate, phenobarbital, levetiracetam phenobarbital, vigabatrin, lamotrigine, clonazepam, clobazam, and gabapentin are choice of drugs for epilepsy in CP children. Polytherapy and monotherapy are compared using the first-line and second-line antiepileptic drugs; it is found that polyepileptic is more effective though full control of the seizures is not always achieved [53–55].

#### 3.7.2. Osteoporosis

Osteoporosis causes weak bones. It causes the bone to break easily with very little stress or a light impact fall. It is often seen in CP patients due to lack of nutrition, decreased weight bearing, and use of certain medicines that causes weakening of the bones. Older patients are advised for screening bone mass before treatment using the fracture risk assessment tool or the Q fracture tool and dual-energy X-ray absorptiometry. Calcium, vitamin D supplements, and bisphosphonates are useful in managing osteoporosis [10, 46]. Weight-bearing exercises are found to improve bone mineral density in cerebral palsy children and hence need to be implemented to improve bone conditions [55].

#### 3.7.3. Behavior Issues

Various behavior disorders are prevalent in CP children. They suffer from conditions such as attention-deficit/hyperactivity disorder, conduct disorders, anxiety, and depression. Cognitive behavior therapy and mental health screening help patients identify and manage behavior issues [10]. “Cool Kids” is a CBT program that is used to manage behavior problems like anxiety and ADHD children. It has been a promising anxiety management program that has been accepted internationally with several clinical trials that have proven to be effective. Experienced psychologists are required to provide the treatment that helps the child and parent develop skills and strategies to manage anxiety and associated impairments [56].

#### 3.7.4. Dysphasia

Swallowing disorders are common in CP children as they occur due to neurological involvement. Its treatment consists of oral care, careful feeding techniques, food modifications, and stimulation of the oral musculature. Drooling occurs in CP children due to weakness of facial muscles and neck muscles. It can be managed with neck posture control, mouth closing, tongue control, behavioral therapies, intraoral appliances, and certain medications like anticholinergic drugs beneficial for this condition. Surgical management includes removal of the salivary glands and duct ligation [57].

#### 3.7.5. Respiratory Problems

Respiratory problems are often seen in CP children which are the main cause of death in adults struggling with this disease. Due to factors such as weakness of the muscles, bad posture, and bad postural control, there is the accidental entry of food particles into the respiratory system which sometimes leads to bacterial growth causing respiratory failures. Management of such conditions includes lifestyle modifications such as postural modifications, food modifications, and weight loss. Improvement of motor functions and respiratory hygiene include improving lung functions, improving lung expansion and aerobic fitness along with airway clearance, and producing effective cough [47]. Oromotor techniques such as sensory awareness training, neck control exercises, general postural management, certain medications, and surgical interventions such as duct transposition and duct ligation are important strategies for managing feeding and swallowing problems [58].

#### 3.7.6. Vision Problems

Abnormal brain development or damage to the brain results in cerebral visual impairment (CVI) previously known as cortical blindness which presents with visual deficits and perceptual deficits. The CVI inventory and assessment are used to diagnose the functional deficits that occur due to cerebral visual impairment [59]. Treatment of hypoxic-ischemic encephalopathy can prevent incidences of CVI, and other treatment approaches like visual stimulation techniques and stem cell therapy need to be assessed further [60]. Vision impairment may be an important aftereffect of brain damage and especially in those born preterm. Some probable aspects that can help these children are family counseling and involving the family in the rehabilitation process and various welfare services from the government like education allowances, special books, scholarship, readers, permission to use assistive devices, large print question paper, scribe for writing examination, extra time in the examination, and substituting visual questions [61].

#### 3.7.7. Sleep Disorders

Sleep disorders are very common in CP children which produces a huge psychological burden on their families. Sleep disorders also cause decreased function. Apart from this, sleep disorders lead to behavioral changes which cause functional problems in the body structure and affect the quality of life of the patient and family. A systematic review was conducted in 2021 to gather information on sleep disorders of CP children under 2 years which found polysomnography as a good assessment technique for CP children. Treatment includes cannabis, surgical interventions, and stimulation of the sensory system [62, 63].

### 3.8. Various Approaches Used in the Management of Cerebral Palsy

#### 3.8.1. Physiotherapy

Physiotherapy has provided great achievement in the field of cerebral palsy. It helps in improving the muscle structure and function and joint range of motion and reduces contractures; some techniques used to achieve this are muscle stretching, joint range of motion exercises, low resistance repetitive exercises, progressive resistance training, functional strength training, balance training, plyometrics, and selective muscle activation by techniques such as constraint-induced movement therapy. A study was done on the effects of neurodevelopmental therapy in CP children which revealed improved function in various activities of children after the application of the intervention technique. NDT also reduced spasticity and improved overall function in CP children; however, there was not much improvement in walking, running, and jumping [8, 10, 17, 21, 64]. Another emergent therapy called hippotherapy has improved neck control and posture control in sitting along with the upper extremity and trunk. There is an overall posture improvement due to stimulation of balance reactions which has a positive effect on balance and spasticity. 30–45 min sessions, twice weekly for 8–12 weeks, produce a positive effect on gross motor function in children with CP [18]. Deep brain stimulation in the case of dyskinetic CP and electrical stimulation via tens and NMES in spastic CP are two techniques to improve the strength and function of muscles [8]. Serial casting is a technique used to stretch tight muscles to improve the range of motion by application of a cast to the affected part [10]. The robot-assisted gait training regimen is effective in improving gross motor function in children whose both sides are affected. There was a positive effect on all the measures of gross motor function after this intervention. It also improved the locomotor ability in ambulatory children [19]. Functional gait training or practice walking on a treadmill with limited body weight support helps in standing erect with a decreased load on the lower extremity joint. This facilitates gait training with good posture and control and is most effective in GMFCS grades IV and V. It can be done with or without a treadmill. Virtual reality and biofeedback can be incorporated; it produces a positive effect [19, 20]. Biofeedback is a common strategy used in rehabilitation that can be used to represent any biological parameters and their changes. The changes can be detected in a variety of ways such as visual, audio, and haptic responses. It is effective in improving motor function by identifying effective motor performance and motor learning [65].

#### 3.8.2. Speech Therapy

There are multiple levels of speech impairment in cerebral palsy children which includes problems such as drooling, swallowing, and feeding having a high rate of 44.0%, 50.4%, and 53.5%, respectively, and as much as half of the children with vertebral palsy are affected with speech problems. Due to the abnormal tone of CP children and impaired musculoskeletal control, speech production and swallowing are difficult in these children. Speech therapy for such conditions helps improve oromotor skills, disarticulation problems, and communication skills [57].

#### 3.8.3. Stem Cell Therapy

Stem cells derived from five areas are present in the literature; they are human umbilical cord blood which is the most common followed by the bone marrow, fetal brain, adipose, and peripheral blood. Autologous stem cells are preferred for children with CP as it shows low immunogenicity. Brain tissue has the maximum amount of neural stem cells, but they have many fewer clinical trials. Peripheral blood cells are highly used stem cells; however, adipose tissue effectiveness is under investigation. Greater improvement was seen in younger children between 10 months and 10 years old. The most efficient route for the administration of stem cells found in the literature is lumbar puncture and intravenous lines; however, it is seen that the prognosis is different in different cases of CP about the administration of stem cells. Apart from this, the effectiveness of the therapy also depends upon the appropriate dose of stem cells. Side effects found in studies with stem cells were fever, nausea, vomiting, and pain at the site of injection, particularly as the lumbar puncture is the preferred method. Hypotonicity is another side effect observed in stem cell therapy using bone marrow. Fine motor function improvement is less compared to gross motor; however, fewer studies have been done in this context [63].

### 3.9. Technological Advances in Cerebral Palsy

#### 3.9.1. Robot-Assisted Devices

Robotics is a novel technique that works on a computerized control system and helps in motor learning and cortical reorganization to improve function in the upper and lower limbs. As functional movements are more fruitful than normal movement patterns, it is found that gait rehabilitation has a more positive impact on lower limb function. With advanced technology, robot-assisted gait training has taken over traditional gait rehabilitation. RAGT is beneficial as it works with increased duration, repetition, constant speed, and pattern. Lower limb robotic exoskeletons are found to be evident in improving the quality of life in CP children. The most evident robotic systems in the literature are Lokomat, Innowalk, Robogait, and Waltbox-K, but due to lack of literature, their efficacy is still a question. It is found that ankle foot orthosis is most beneficial for lower limbs in CP children, and an electronic variance of such device is scarce. There are many clinical trials, but review studies are lacking in the literature. Extensive studies are required for upper limb robotic assistance. Apart from these, social robots are also another milestone in artificial intelligence that has to improve communication and participation among CP children along with motivation in rehabilitation [66, 67]. Some of such major technological advances are described in Table 8.

#### 3.9.2. Virtual Reality

Virtual reality is a recent development in the field of neurorehabilitation that induces imaginations as real as reality, and patients are allowed to perform functional activities in such environments. It was developed in the 1960s and is used as a diagnostic tool in certain psychiatric cases. Many clinical trials are being conducted on VR among stroke patients, COPD patients, and most recently in obstetric and gynaec cases. Though VR is used as a pain management strategy in CP patients, functional outcome studies are scarce in this population [68].

#### 3.9.3. Augmentative and Alternative Communication Devices

The communication problem is found to be present in 25% of cases of cerebral palsy children, and most of this population has some or other oromotor problems. Augmentative and alternative communication devices are used to improve the communication abilities of speech-impaired children. It helps develop a communication pattern among the CP child and various other members of the community. There are certain manual boards used in this strategy that can be used in the form of figures, number symbols, etc. Other AAC devices used with speech and language problems are some form of technological device that helps to expose the thinking of the child. All studies with AAC shows a good result, but very less studies are done on CP children [69].

#### 3.9.4. Mobile Applications for Cerebral Palsy Children

The use of mobile applications has drastically changed the scenario of the health care delivery system. These apps are reliable and valid and have become very common and handy that are beneficial in transferring information, forming analysis, and monitoring and treatment. 23 mobile applications are specifically used for cerebral palsy children and many others that can be of importance. Some services of these apps are correcting foot deformities of CP children by producing an auditory signal during altered biomechanics in foot placement and risk assessment of hip dysplasia in CP children by health care specialists [25].

## 4. Conclusion

Injury to the developing brain before, during, and after birth causes various symptoms to evolve in a child; the condition is referred to as cerebral palsy. It affects the normal movement in different parts of the body along with problems such as abnormal resistance to movements, the attitude of the body, and movement and activity limitation, accompanied by various sensory disturbances along with perception, cognition, communication, behavior, epilepsy, and secondary musculoskeletal problems. The causes may be prenatal, perinatal, and postnatal. Certain risk factors are identified for damaging the brain that also include the health condition of the mother before conception. The prevalence of CP is found to be stable in various epidemiological studies owing to different preventative, neonatal care, and postnatal strategies. Brain damage during the developing period or immediately after birth can be detected by gathering information from the mother during events that have occurred before or during delivery and certain early signs in the child. Diagnostic tools are now available for the detection of cerebral palsy as early as less than 5 months. Based on the heterogeneity of the condition, various associated problems arise in the child along with motor disturbances which need to be assessed and managed by a multidisciplinary team of specialists. Along with the traditional management approaches of cerebral palsy, many emerging techniques are making advancements in research that have future scopes of advancement to give a pleasant and functional life to the affected children. A new view of cerebral palsy etiology is the recent development of genetic studies on cerebral palsy as there are cases found which have no prenatal, natal, or postnatal explanations. Research on these areas may be beneficial to find a correlation between genetics and cerebral palsy. Apart from this, artificial intelligence also directs future study and rehabilitation strategies to improve the functional status of cerebral palsy children.

## Figures and Tables

**Table 1:** Risk factors for cerebral palsy [2, 7, 10].

Preconception	Before birth	During birth	After birth
Systemic illness of the mother	Premature birth	Premature birth	Hypoxic ischemic encephalopathy
Use of drugs and stimulants	Low birth weight	C-section	Infection
Immune system disorders preceding pregnancy	CNS malformation	Vacuum-assisted delivery	Hyperbilirubinemia
Spontaneous abortions	Maternal DM	Delivery after the due date	Cerebrovascular accidents
Socioeconomic factors	Prolonged rupture of membrane	Prolonged labor	Intracranial hemorrhage
Poisoning	Maternal hemorrhage	Asphyxia	CNS infection
Infections	Multiple gestations	Meconium aspiration	Respiratory distress syndrome
Impaired fertility	Cotwin death	Breech vaginal delivery	Artificial respiratory support
Treatment of fertility	Genetic factors	A high fever during delivery	Hypoglycemia neonatal convulsions
Genetic factor	Encephalopathy of prematurity	Perinatal stroke	Traumatic brain injury
	Congenital malformation		Near drowning
	Hypoxic ischemic encephalopathy		Meningitis
	In utero stroke		Sepsis
	In vitro fertilization		Neonatal encephalopathy
	Kernicterus		
	Maternal disorder of clotting		
	Meconium aspiration		
	Fetal growth restriction		
	Preeclampsia		

**Table 2:** Etiologies of cerebral palsy [2, 8, 11–13, 15].

Prenatal	Perinatal	Postnatal
Infection and fever during pregnancy	Obstructed labor	Hypoglycemia
Metabolic disorders	Cord prolapses	Jaundice
Intrauterine infection	Antepartum hemorrhage	Neonatal meningitis
Chorioamnionitis	Metabolic acidosis	Septicemia
Maternal ingestion of toxins	Use of assisted reproductive technology	Malaria
Preeclampsia	Intrapartum hypoxia	Malaria with seizures
Maternal trauma in pregnancy		Malaria with coma
Exposure to methylmercury Genetic syndromes Multiple pregnancies		Meningitis
IUGR		Tuberculosis
Fetal growth restriction		Sickle cell disease
Placenta abruption		HIV
Failure of closure of the neural tube		PVL
Schizencephaly		Congenital infections
Chromosomal defects		Asphyxia
Microcephaly		Hyperbilirubinemia
Rubella		Genetic causes
		Neonatal stroke

**Table 3:** SCPE classification of cerebral palsy [2, 13].

Type of CP	Description
Spastic	Presents with hypertonicity and hyperreflexia
	May be unilateral or bilateral
	Presents with involuntary, uncontrolled, repetitive, and sometimes stereotype movements with altered muscle tone
Dyskinetic	

	Abnormal posture with hypertonicity is termed dystonic
	A quick, uncontrolled, and twisting movement with hypotonia is called choreoathetosis
Ataxic	In coordination with a decreased muscle tone

**Table 4:** Functional classification of children with cerebral palsy [2, 11, 12, 23].

Classification type	Description
GMFCS	Evaluates the gross motor function of the individual with CP
MACS	Evaluates functions of upper limb
CFCS	Evaluates everyday communication
EDACS	Evaluates the ability to eat for children with CP after 3 years

**Table 5:** Early signs of cerebral palsy [22].

Early signs of CP
Early hand dominance
Delayed motor milestones
Persistent primitive reflexes
Scissored legs below 6 months
Floppiness
Stiffness

**Table 6:** Comorbidities associated with cerebral palsy [11, 17–20].

Comorbidities present in CP children	
Pain	75%
Intellectual disability	50%
Gait disorders	33%
Hip displacement	33%
Speech problems	25%
Epilepsy	25%
Incontinence	85%
Behavior disorders	25%
Sleep disorders	40%
Hearing impairment	9%
Vision impairment	10%
Cognitive impairment	77%
Thyroid dysfunction	3%
G.I. disturbances	2%

**Table 7:** Tools for early diagnosis of CP [4, 13].

Below 5 months	Above 5 months
GMA	Magnetic resonance imaging
MRI	
HINE	The Hammersmith Infant Neurological Examination
Prechtl's Qualitative Assessment of General Movements	The Developmental Assessment of Young Children
The Hammersmith Infant Neurological Examination	

**Table 8:** A statistical analysis of technological advancement in cerebral palsy management from 2016 to 2022.

Author (year)	Title	Summary
A Shierk et al. (2016)	Review of therapeutic interventions for the upper limb classified by manual ability in children with cerebral palsy	There was some form of improvement in the hands using various intervention techniques but only in MAC levels II and III.
		Extensive studies are required in levels IV and V [70].

Atefehaboutorabi et al. (2017)	Efficacy of ankle foot orthoses types on walking in children with cerebral palsy: a systematic review	Specific types of orthosis improve ankle and knee range of motion, walking speed, and stride length in CP children. The reduced energy expenditure was found to be effective in improving stride length, speed of walking, single limb support, and gait symmetry; it also helped in decreasing energy expenditure of hemiplegic CP as compared with the barefoot condition. Further studies are required for better evidence regarding this [71].

Anna Alves Pinto (2016)	The case for musical instrument training in cerebral palsy for neurorehabilitation	The study inferred that playing musical instruments may help produce plastic changes in the brain for developing skills of CP children [72].

Adam T C Booth et al. (2018)	The efficacy of functional gait training in children and young adults with cerebral palsy: a systematic review and meta-analysis	Functional gait using a treadmill with little body weight support helps present an upright posture and improves gait. The authors suggested that virtual reality and biofeedback improve function [73].

Ali Reza Jamali (2018)	The effects of constraint-induced movement therapy on functions of cerebral palsy children	The therapeutic effect of CIMT is independent of age and gender, but its effect on muscle tone and protective extension is yet to be investigated [48].

Atefehaboutorabi et al. (2017)	Efficacy of ankle foot orthoses types on walking in children with cerebral palsy: a systematic review	Specific types of orthosis improve ankle and knee range of motion, walking speed, and stride length in CP children. They reduce energy expenditure and were found to be effective in improving stride length, speed of walking, single limb support, and gait symmetry; it also helped in decreasing energy expenditure of hemiplegic CP as compared with the barefoot condition. Further studies are required for better evidence regarding this [74].

Alexendermaclntosh et al. (2019)	Biofeedback interventions for individuals with cerebral palsy: a systematic review	Biofeedback interventions will help improve movement patterns in cerebral palsy children; however, poor quality and quantity studies are hindering finding the actual efficacy of the technique [65].

Ana Paula Salazar et al. (2019)	Neuromuscular electrical stimulation to improve gross motor function in children with cerebral palsy: a meta-analysis	NMES improves gross motor function in children with CP. However, it was found effective to improve GMFM-sitting and standing dimensions but not GMFM-walking dimensions; however, the literature found was of low quality [75].

Anna Tevelde (2019)	Early diagnosis and classification of cerebral palsy: an historical perspective and barriers to an early diagnosis	A timeline of calls for early diagnosis is described in the paper. Reduction of age for diagnosis and factors that cause difficulty in diagnosis are evaluated [76].

Cihanuyanik et al. (2022)	Brainy home: a virtual smart home and wheelchair control application powered by brain-computer interface	BCI is being used as a smart technology in normal settings; however, it is very rarely used in disabled populations. This study is done in a virtual setup among the disabled population and reveals that it will be an effective means of improving communication and functional status among this population shortly in the real world [77].

David Graham (2019)	Current thinking in the health care management of children with cerebral palsy	This paper focuses on early diagnosis and treatment to be an effective management strategy for CP. It also discusses various techniques of management like oromotor stimulation, deep brain stimulation, and functional classification of CP children [37].

Ewelina Matusiak-Wieczorek et al. (2019)	The influence of hippotherapy on the body posture in a sitting position among children with cerebral palsy	Hippotherapy has a positive effect on head position, arm function, and trunk control in mild cerebral palsy children. The study concluded that hippotherapy has a positive influence on body posture and the function of different body structures in sitting positions [78].

Eli Kinney-Lang et al. (2022)	Advancing brain-computer interface applications for severely disabled children through a multidisciplinary national network: summary of the inaugural pediatric BCI Canada meeting	BCI helps children with disabilities to communicate with their thoughts, and those who are cognitively sound children can benefit from this new technology. However, as this is a young field, very fewer studies are done, so it is recommended that various researches using BCI shall be conducted in different populations and different geographical locations [79].

Hussein ZA et al. (2019)	Effect of simultaneous proprioceptive-visual feedback on the gait of children with spastic diplegic cerebral palsy	A 3 times/week treatment for 2 months with simultaneous proprioceptive and visual feedback resulted in significant differences in spatial and temporal parameters of gait; however, there were fewer effects on kinetic gait parameters [80].

Hongyuchen et al. (2016)	A review of wearable sensor systems for monitoring body movements of neonates	The study focuses mainly on the use of wearable sensors for body movements for detecting body movements in very young children as movements in babies give an idea about the level of brain development and brain damage [81].

Irene Mall et al. (2017)	Functional electrical stimulation of the ankle dorsiflexors during walking in spastic cerebral palsy: a systematic review	FES can be a helpful adjunct as a replacement for orthosis in lower limbs of cerebral palsy children though it needs extensive studies in the future for improving the state of spastic muscles [82].

Iona Novak et al. (2017)	Early, accurate diagnosis and early intervention in cerebral palsy: advances in diagnosis and treatment	Diagnosis and prediction of cerebral palsy age have been reduced from 12 months to as low as 5 months using various advances like HINE, PQAGM, and MRI [83].

Jakub Mlodawsk et al. (2019)	Cerebral palsy and obstetric-neonatological interventions	Various strategies to prevent brain damage before birth were discussed along with a focus on risk factors during delivery and after delivery. Indications of hypothermia are explained in detail [84].

Jing Zhang (2017)	Multivariate analysis and machine learning in cerebral palsy research	This study briefs out the use of machine learning and multivariate analysis in predicting developing brain damage. Its future implications include detecting and management of cerebral palsy using the same [85].

Joao Pedroproenka (2017)	Serious games for upper limb rehabilitation: a systematic review	The study found that computer games are an emerging technique to improve upper limb function in cerebral palsy children; however, extensive search is required to improve its efficacy [86].

Jessica Rose et al. (2017)	Artificial walking: technologies to improve gait in cerebral palsy: multichannel neuromuscular stimulation	The study focuses on the use of neuromuscular electrical stimulation as it has much more benefits than traditional medical and surgical techniques to improve the walking pattern of hypertonic CP children [87].

Jun Wang et al. (2018)	Effect of suspension exercise training on motor and balance functions in children with spastic cerebral palsy	Motor functions and balance improve with suspension exercise training in plastic cerebral palsy [88].

Jyoti Upadhyay (2020)	Cerebral palsy: aetiology, pathophysiology, and therapeutic interventions	Common causative agents of cerebral palsy before, during, and after delivery are discussed, and the use of a new technology called electrical stimulation to improve muscle strength along with deep brain stimulation is on the verge of therapy for CP patients [8].

Li Hua Jin et al. (2020)	The effect of robot-assisted gait training on locomotor function and functional capability for daily activities in children with cerebral palsy: a single-blinded, randomized cross-over trial	RAGT improves walking ability and improves the activities of daily living. Better effects were seen in children who can walk with support [89].

Michael T Clarke et al. (2016)	Augmentative and alternative communication for children with cerebral palsy	This study throws light on the use of AAC strategies using sign language and various other ways to improve communication and language among speech and learning impaired cerebral palsy children [20].

Masahito Mihara et al. (2016)	Review of functional near-infrared spectroscopy in neurorehabilitation	This review concluded that NIRS is an emerging investigation tool and needs further follow-ups to use it as a therapeutic modality [90].

Mary M Rodgers et al. (2019)	Wearable technologies for active living and rehabilitation: current research challenges and future opportunities	Wearable technologies are being used to improve functional status. However, it is used for a short period in all the evident studies. This study urges to find the efficacy of these devices when used for a longer duration. There are certain barriers in this context such as the ability to use and comfort status of the wearable [91].

Mahindra Rana et al. (2017)	A systematic review on etiology, epidemiology, and treatment of cerebral palsy	Various classifications of CP are discussed. Factors that lead to brain damage and their prevention along with recent technological advances are focused on [14].

Moshe Stavsky et al. (2017)	Cerebral palsy—trends in epidemiology and recent development in prenatal mechanisms of disease, treatment, and prevention	The occurrence of CP has been stable for the last two decades, and the paper has discussed various strategies to prevent brain damage in the developing period. They also discussed the effects of various recent techniques [7].

Neha A. Parikh et al. (2019)	Early detection of cerebral palsy using sensorimotor tract biomarkers in very preterm infants	The use of biomarkers to evaluate the presence of brain damage is an effective technique as discussed in this paper [5].

Peter Wilson et al. (2016)	Integrating new technologies into the treatment of CP and DCD	The study discussed the effect of advanced techniques like virtual reality and its impacts on developmental disorders like CP and DCD [92].

Petra Karlsson (2022)	Brain-computer interface is a potential access method for communication in non-verbal children with cerebral palsy: a state-of-the-art review	This study focuses on the fact that BCI is a promising technology that is emerging very rapidly in the literature, and cerebral palsy children will be largely benefited from this technological advancement.

Qi Wang et al. (2017)	Interactive wearable systems for upper body rehabilitation: a systematic review	Wearable systems are important for various neurological disorders. They use sensors, accelerometers, and inertial measurement units for measuring improvement parameters in the upper limb [92].

Rocco Salvatore Calabro et al. (2016)	Robotic gait rehabilitation and substitution devices in neurological disorders: where are we now	This study has discussed in detail various robotic rehabilitation techniques that are used for various neurological cases including cerebral palsy to improve their walking abilities [93].

Shahshanchen et al. (2016)	Toward pervasive gait analysis with wearable sensors: a systematic review	This paper focuses on wearable sensors for the evaluation of gait kinetics and kinematics as the existing technologies like OGA and force plates are costly and need expertise [94].

Sonika Agarwal et al. (2021)	Cerebral palsy and rehabilitative care: the role of home-based care and family-centered approach	Home-based physiotherapy programs, telemedicine, and video monitoring of home-based therapies are found to be effective among these children [95].

Stanislava Klobucká et al.	Effect of robot-assisted gait training on motor functions in adolescent and young adult patients with bilateral spastic cerebral palsy: a randomized controlled trial	The robot-assisted gait training regimen is more effective than conventional therapy in terms of improvements in gross motor functions in adolescent and adult patients with bilateral spastic CP [96].

Tony W. Wilson et al. (2016)	Neuroimaging with magnetoencephalography: a dynamic view of brain pathophysiology	This study focuses on the use of MEG in processing neural information of the brain and to find out the abnormal neural information processing in cerebral palsy cases along with other neurological cases. It also implicates future studies in this context [97].

Wei-Peng Teo (2016)	Does a combination of virtual reality, neuromodulation, and neuroimaging provide a comprehensive platform for neurorehabilitation? – A narrative review of the literature	The study focuses on the use of VR in combination with various other recent technologies for improving its effect on CP children. The study urges larger studies in the same context [98].

Yupigchen et al. (2018)	Effectiveness of virtual reality in children with cerebral palsy: a systematic review and meta-analysis of randomized controlled trials	The review found VR to be an effective technology in comparison to other techniques to improve movement in cases of brain damage [68].

Chen and Howard (2016)	Effects of robotic therapy on upper-extremity function in children with cerebral palsy: a systematic review	Various components of hand function improved using the robotic therapy; however, more studies are required with larger cohorts [99].

Zeannajadavji et al. (2021)	Can children with perinatal stroke use a simple brain-computer interface?	BCI is an emerging and promising technology that can help individuals with brain damage. Future studies are directed toward the effect of BCI among unilateral stroke due to early brain damage [100].

Zhong-Yue Lv et al. (2020)	Progress in clinical trials of stem cell therapy for cerebral palsy	Stem cells from human umbilical cord blood are the most common followed by bone marrow, fetal brain, adipose, and peripheral blood. Autologous stem cells are preferred for children with CP. Greater improvement was seen in younger children between 10 months and 10 years old. Lumbar puncture and intravenous injection are mostly used to insert the stem cells. Gross motor function and cognition abilities improve better with this treatment [101].

## Data Availability

The data underlying the results presented in the study are available within the manuscript.
